# Endovascular intervention in renovascular disease: a pictorial review

**DOI:** 10.1007/s13244-014-0363-z

**Published:** 2014-10-12

**Authors:** Jagbir Khinda, Sriharsha Athreya

**Affiliations:** 1 Department of Medical Imaging, Faculty of Medicine, University of Toronto, 263 McCaul Street-4th Floor, Toronto, Ontario M5T 1W7 Canada; 2 Michael G. Degroote School of Medicine, McMaster University, Hamilton, Ontario Canada; 3 Department of Radiology, St. Joseph’s Healthcare Hamilton, 50 Charlton Ave. E., Room T0112, Hamilton, ON L8N 4A6 Canada

**Keywords:** Endovascular procedures, Renal artery obstruction, Hypertension, Renovascular, Aneurysm, Arteriovenous malformations

## Abstract

Interventional radiologic procedures offer a significant and expanding role in the management of various renovascular diseases including renal artery stenosis, renal artery aneurysm and pseudoaneurysm, renal vascular malformations, renal tumours, trauma, and resistant hypertension. In this article, we discuss these entities in the context of currently accepted definitions, incidence, modes of diagnosis, and management as they pertain to the practice of interventional radiology. Particular emphasis is placed on current interventional procedures for managing and treating these diseases as well as emerging procedures and technologies.

• *Highlights the literature on renovascular diseases*

• *Reviewing the role of various interventional procedures in the management of renovascular disease*

• *Review of imaging techniques in the identification and characterisation of renovascular disease*

## Introduction

The role of interventional radiology in disease management is rapidly advancing. With ongoing innovation, interventional procedures are playing a growing role in the treatment of various renovascular diseases. This article highlights the literature on renovascular diseases with an emphasis on the current endovascular procedures for managing and treating these diseases as well as emerging procedures and technologies.

## Renal artery stenosis

Renal artery stenosis (RAS) is a narrowing of the renal arteries, often associated with renovascular hypertension and an increased risk of renal insufficiency. Atherosclerotic RAS constitutes 90 % of RAS cases, increasing in prevalence in patients with diabetes, hypertension, aortoiliac occlusive disease, and coronary artery disease, as well as with age [1]. Fibromuscular dysplasia (FMD) accounts for fewer than 10 % of RAS cases. FMD occurs nine times more frequently in females than in males [2], tending to affect women aged 15 to 50 [1].

Numerous physiologic and imaging-based modalities exist for the evaluation of patients with clinical suspicion of RAS [1]. Generally, noninvasive evaluations by duplex ultrasonography, magnetic resonance angiography (MRA), or computed tomographic angiography (CTA) serve as initial diagnostic tools [1]. Subsequent catheter-based angiography permits confirmation of the RAS, aetiology, extent of intrarenal vascular disease, as well as intervention planning [1, 2]. Contrast medium must be used cautiously in patients with renal failure given the risk of contrast-induced nephropathy. Digital subtraction techniques may decrease the required contrast volume, thereby minimising impact on renal function [1].

**Fig. 1:**
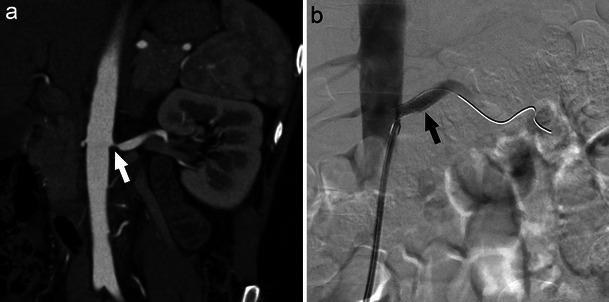
CT angiography demonstrating significant left proximal main renal artery stenosis (**a**, white arrow) managed with percutaneous transluminal renal angioplasty and stent placement (**b**, black arrow)

**Fig. 2:**
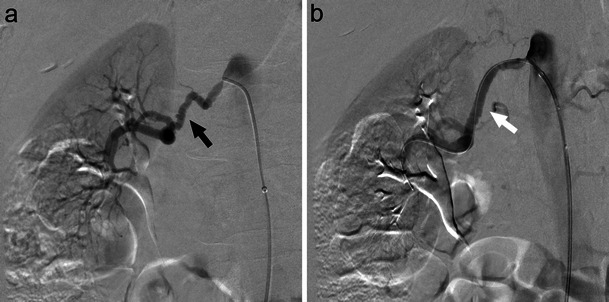
Beaded, aneurysmal appearance of the distal renal artery secondary to FMD. Secondary hypertension due to renal artery stenosis in this case was effectively managed with balloon angioplasty (**b**, white arrow)

RAS resulting from atherosclerotic disease typically involves the ostium and proximal third of the main renal artery (Fig. 1a). FMD refers to a collection of vascular diseases of the intima, media (90 %), and adventitia (periarterial) [1]. RAS secondary to medial FMD is characterised by a beaded, aneurysmal appearance localised to the distal two-thirds of the renal artery and its branches (Fig. 2a), whereas intimal and periarterial FMD is commonly associated with progressive vessel dissection and thrombosis [1]. Bilateral renal artery FMD is observed in greater than 35 % of patients [2, 3].

The role of percutaneous transluminal renal angioplasty (PTRA) in the treatment of renovascular hypertension caused by RAS is debatable. While improvement or cure of hypertension is consistently shown in 70–98 % of FMD-induced hypertension cases [2], the success of atherosclerotic RAS angioplasty remains unclear. Retrospective and registry analyses have demonstrated enhanced blood pressure control and improved or stabilised renal function following RAS angioplasty [4–6]. Despite these findings, several randomised trials have consistently failed to reflect similar clinical benefit—both with and without stenting—when compared to medical therapy alone [4, 7–9]. Furthermore, while demonstrating no clear clinical benefit with regards to renal function, blood pressure, renal or cardiovascular events, or mortality, angioplasty carried substantial risk, including pulmonary oedema, myocardial infarction, renal embolisation, and renal artery perforation [9].

Nonetheless, many experts continue to advocate for PTRA in specific cases of RAS, such as resistant hypertension, haemodynamically significant atherosclerotic RAS (Fig. 1b), FMD-associated RAS (Fig. 2b), deteriorating renal function, bilateral atherosclerotic RAS, tight atherosclerotic RAS in a single kidney, unexplained CHF, and recurrent episodes of flash pulmonary oedema [4, 10].

## Renal artery aneurysm

**Fig. 3:**
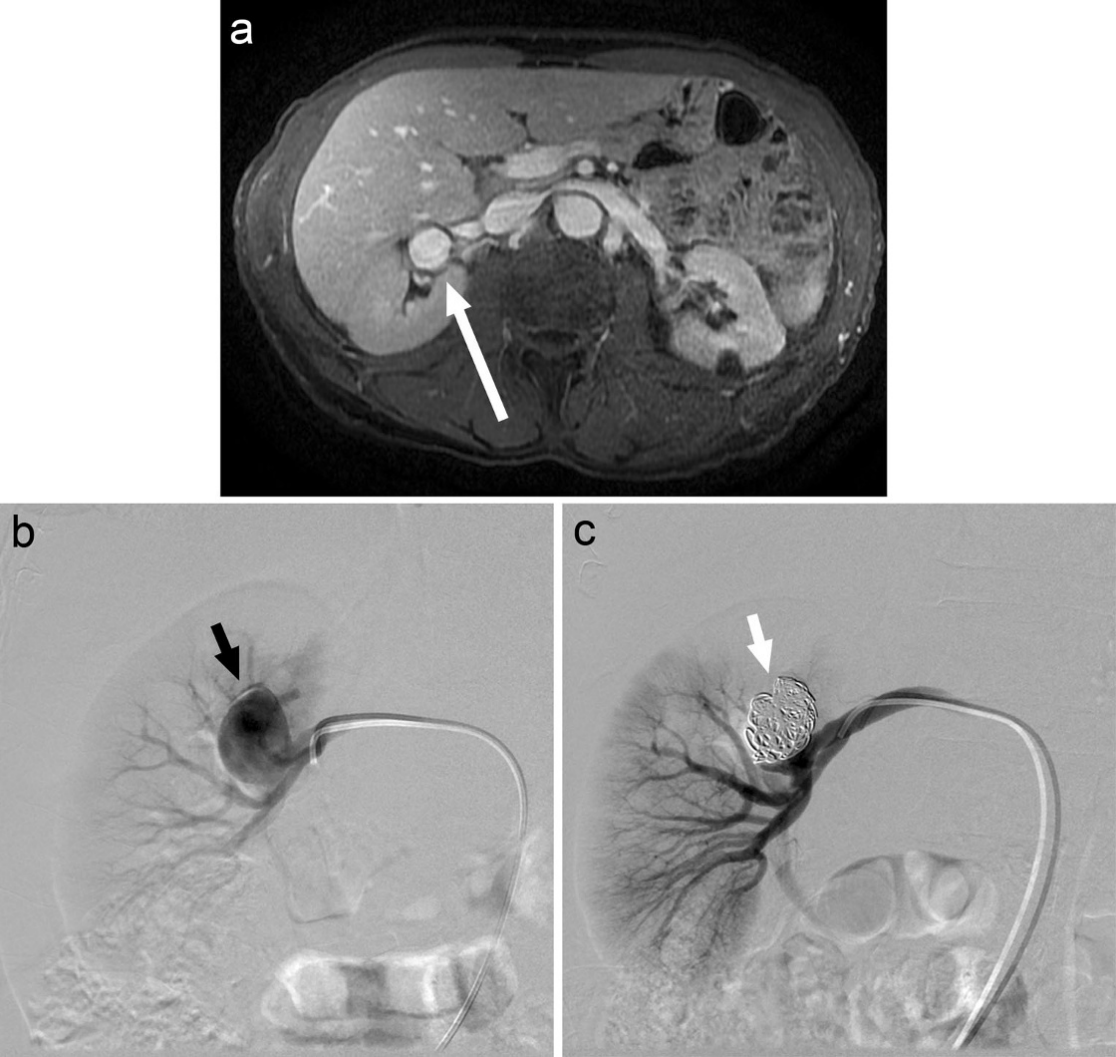
A true extraparenchymal saccular right renal artery aneurysm seen on MR (**a**, long white arrow) and angiography (**b**, black arrow) effectively managed with intraaneurysmal coil embolisation (**c**, white arrow)

Renal artery aneurysm (RAA) is a dilatation of a segment of the renal artery exceeding one and a half times the normal diameter [11]. RAAs are relatively uncommon (prevalence <0.1 %), often identified incidentally on MRI or CT imaging studies (Fig. 3a) [12]. RAAs may be asymptomatic or progress to cause hypertension (most common finding), haematuria, and flank pain in cases of rupture [13, 14]. Classification exists on the basis of location, aetiology, and morphology of the aneurysm. The majority of RAAs are extraparenchymal, 70 % of which are saccular lesions. Fusiform and dissecting RAAs account for the remainder of extraparenchymal lesions while only 15 % of RAAs are intraparenchymal [15, 16].

True aneurysms are saccular or fusiform in morphology, encompassing all layers of the vascular wall. Generally, these lesions are secondary to underlying inherited disorders such as FMD and Ehlers-Danlos syndrome [16].

Indications for therapeutic intervention of RAAs include rupture, diameter >2.0 cm, renovascular hypertension, dissection, rapid expansion on serial imaging, localised symptoms (haematuria), and distal embolisation impairing renal function [11]. While uncommon (5.6 % incidence), mortality rates from RAA rupture may be as high as 80 % [14]. Pregnancy carries a high risk of rupture—80 % in women with pre-existing RAA—and thus some studies suggest treatment of RAAs in all women of child-bearing age [14, 15, 17].

Aneurysm location, morphology, and size; branches arising; artery of involvement; condition of the artery; age; patient health; and renal function are all determinants in guiding endovascular treatment (Fig. 3b, c) [13, 16]. Table 1 outlines RAA anatomic considerations and treatment guidelines. Recent case reports and small studies have demonstrated satisfactory visceral aneurysm thrombosis and shrinkage (including RAA) by flow-diverting devices. These stents function to reduce flow velocity within the aneurysm sac and promote thrombosis while maintaining main artery and branch vessel flow [18].

**Table 1:** Renal artery aneurysm classification and management

	Definition	Management
Type 1	Saccular Main renal artery or large segmental branches [17]	
Narrow neck	Neck opening <70%^a^ [27]	Intraaneurysmal coil embolisation [27, 45]
Wide neck	Neck opening ≥70 % ^a^ [27]	BMS with intraaneurysmal coil embolisation [20, 27] Stent grafting [45]
Bifurcation	Located at bifurcation [20]	Y-shaped stent grafting [16] Combination stent grafting and embolisation [12, 16]
Type 2	Fusiform Main renal artery or proximal large segmental arteries [13, 16]	Surgical [15] Covered stent grafting [16]
Type 3	Intralobar, intraparenchymal [16]	Embolisation of end arteries [16, 19]

BMS, Bare metal stent. ^a^In comparison to aneurysm diameter

Complications of the endovascular intervention in RAA include non-target embolisation (potentially resulting in renal failure or loss secondary to infarction), worsening hypertension, stent thrombus, infection, and radiation skin burns [16]. Available short-term data have demonstrated promising findings with respect to safety, successful aneurysm exclusion, and improvement in hypertension and renal dysfunction [19]. Unfortunately, studies analysing the long-term efficacy of endovascular RAA intervention are currently lacking.

## Renal artery pseudoanerysms

Unlike true aneurysms, pseudoaneurysms are periarterial haematomas resulting from a tear of the arterial wall [20]. Typically, pseudoaneurysms are acquired because of blunt or penetrating abdominal trauma, iatrogenic injury, or inflammation, and they possess subsequent potential for enlargement and/or rupture [20, 21]. These rare lesions are often saccular in appearance and may be either intraparenchymal or extraparenchymal.

Due to a propensity for severe haemorrhage, early detection and management, particularly in haemodynamically unstable patients, are essential. CT angiogram provides the mainstay in diagnosis [21]. Subsequent angiography offers the benefit of guiding the intervention, allowing for target vessel localisation and assessment of inflow and outflow vessel size prior to management.

**Fig. 4:**
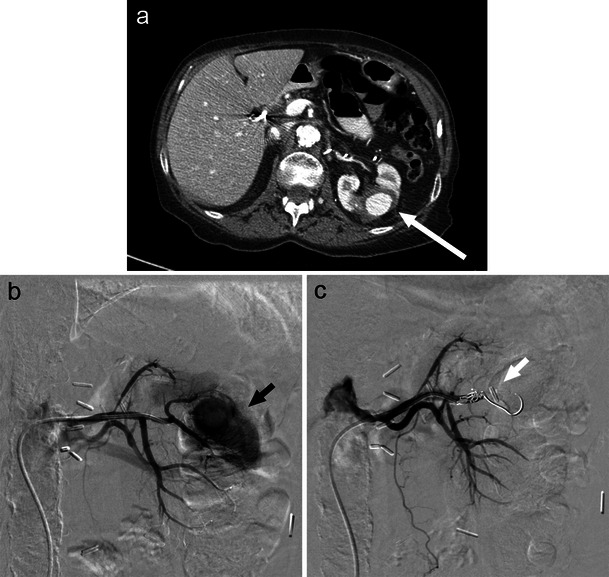
Axial CT (**a**, long white arrow) and angiographic (**b**, black arrow) images of a left intraparenchymal renal artery pseudoaneurysm subsequent to partial nephrectomy for renal tumour. The propensity for severe haemorrhage with these lesions warranted selective coil embolisation (**c**, white arrow)

Currently, selective coil embolisation of the affected artery is the accepted treatment modality for most intraparenchymal renal pseudoaneurysms (Fig. 4) [20, 21]. Extraparenchymal aneurysms of larger vessels may often be managed using covered stents without causing renal infarction. Studies have demonstrated endovascular management of renal artery pseudoaneurysm to be both effective and safe, rarely resulting in long-term renal impairment [21]. Complications of embolisation are similar to those discussed earlier.

## Renal arteriovenous malformations and fistulas

Renal arteriovenous malformations (RAVMs) and renal arteriovenous fistulas (RAVFs) represent arteriovenous communications of the renal vasculature. An RAVM is a congenital network of abnormal vessels characterised as cavernous (single arterial feeder) or cirsoid (multiple arterial feeders with corkscrew appearance) in nature [16]. Large-scale autopsy studies have indicated the prevalence of RAVMs to be approximately 1 in 30,000, with cirsoid-type RAVMs occurring more frequently than cavernous types [16, 22].

An RAVF represents a direct communication between an artery and a vein [16, 23]. Greater than three-quarters of these lesions are acquired, often resulting from iatrogenic causes such as renal biopsy, percutaneous nephrostomy, or accidental trauma [16].

**Fig. 5:**
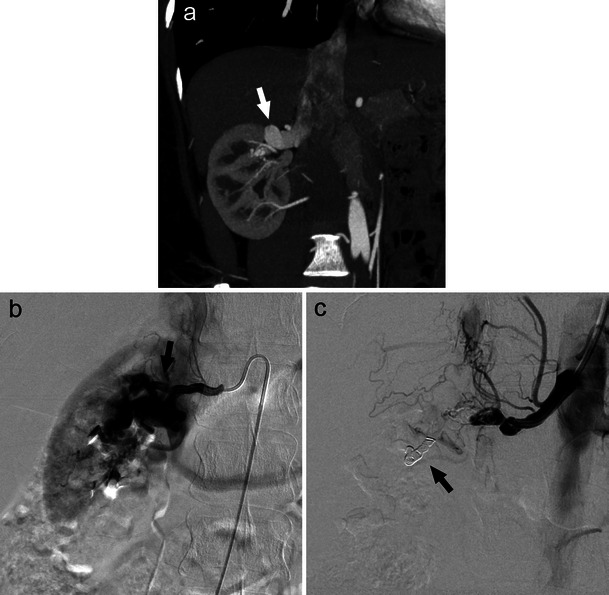
Right RAVM as seen on CT (**a**, white arrow) and angiogram (**b**, black arrow). Coil embolisation provides an effective option in the treatment of larger RAVMS (**c**, black arrow)

**Fig. 6:**
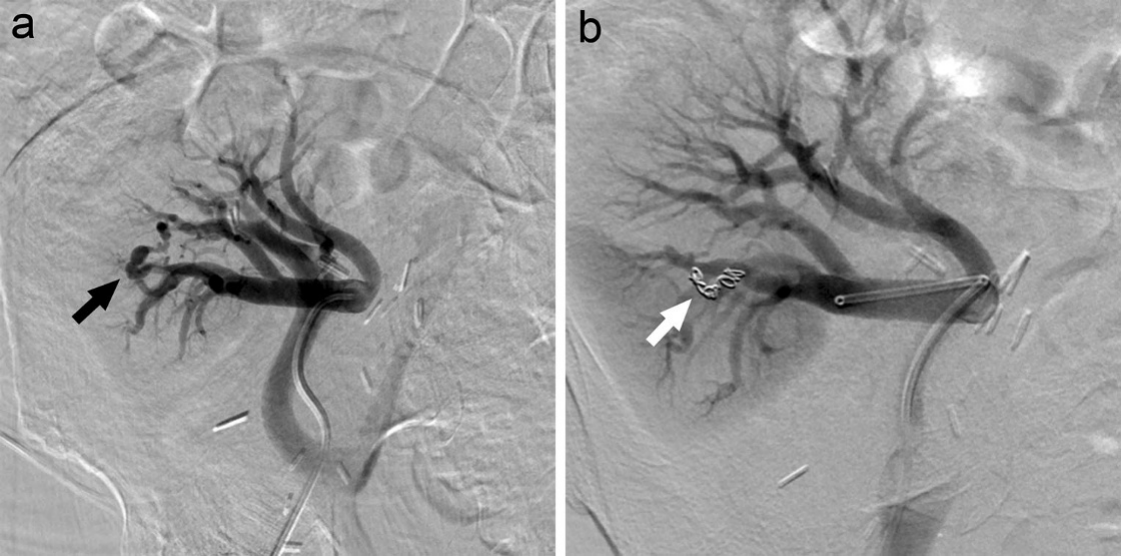
Angiographic image of a right RAVF (**a**, black arrow) developed following ultrasound-guided biopsy of a transplant kidney with subsequent selective coil embolisation of arterial feeders (**b**, white arrow)

These renovascular abnormalities are often identified during workup for gross haematuria, flank pain, and hypertension [16]. Colour Doppler US, CT, and contrast-enhanced MRA are all useful imaging modalities in the diagnosis of these malformations (Fig. 5a, b; Fig. 6a) [16]. Digital subtraction angiography (DSA) demonstrates the detailed vascular anatomy and is typically performed prior to embolisation.

Pain, persistent haematuria, cardiac failure, and hypertension are all indications for potential interventional treatment of RAVMs [16]. First-line management of RAVMs typically consists of malformation destruction and arterial occlusion with sclerosing agents such as absolute alcohol. These agents stimulate thrombus formation through toxic effects on the endothelium, arterial spasm, and perivascular necrosis [16]. Autologous clot, polyvinyl alcohol, gelatin sponge, and n-butyl cyanoacrylate glue are also proven embolic agents in this setting [24]. Selective coil embolisation of arterial feeders may be useful in the treatment of larger RAVMs (Fig. 5c) and RAVFs as well as to rapidly decrease blood flow in acute haemorrhage (Fig. 6b) [16, 23].

Transcatheter embolisation (TCE) as treatment for RAVMs and RAVFs is considered safe and effective with studies demonstrating excellent technical success (100 %) and resolution or improvement in clinical symptoms [25, 26]. Risks include non-target infarction, toxic effects from entry of absolute alcohol into the systemic vasculature, and coil migration to non-target/venous circulation [16, 27].

## Renal tumours

**Fig. 7:**
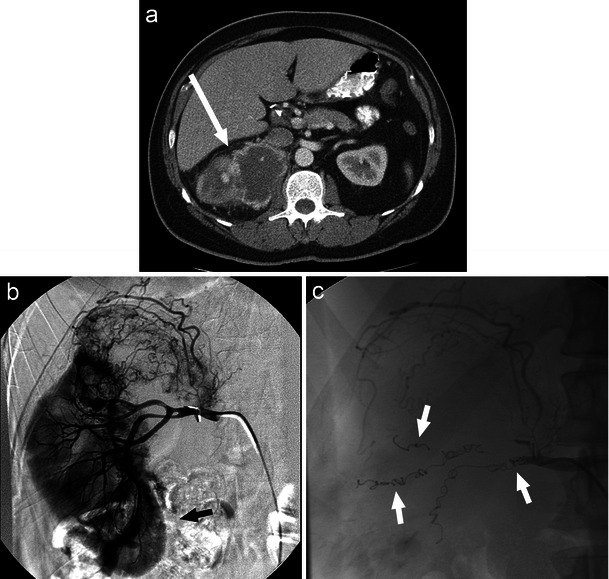
A large right RCC as demonstrated on CT (**a**, long white arrow) and angiographic imaging (**b**, black arrow). In this case, renal artery TCE (**c**, white arrows) allowed for tumour devascularisation prior to surgical resection

Renal cell carcinoma (RCC) is the most common primary renal malignancy, accounting for over two percent of all adult cancers [28]. Dedicated renal CT (Fig. 7a) and MRI provide optimal imaging for the characterisation of renal tumours with comparable accuracy. Ultrasound may also play a role in early investigation and characterisation of renovascular anatomy [28, 29].

Renal artery transcatheter embolisation serves a variety of roles in RCC management (Fig. 7b, c). Preoperatively, TCE is useful in devascularisation to ease surgical resection and minimise blood loss. In the palliative setting, RCC TCE may provide symptom relief from haematuria and flank pain through reduction of tumour size [28, 30]. Technical success may be achieved with a variety of embolic agents. Ethanol, polyvinyl alcohol, and embospheres are often used for renal parenchyma capillary occlusion with subsequent coil embolisation of the main renal artery and any extrarenal feeding vessels [30]. Controversy exists regarding the long-term benefit of pre-operative embolisation with one study reporting increased 5-year survival benefit over patients who undergo nephrectomy alone [31, 32], while others demonstrated no long-term survival benefit [31, 33].

**Fig. 8:**
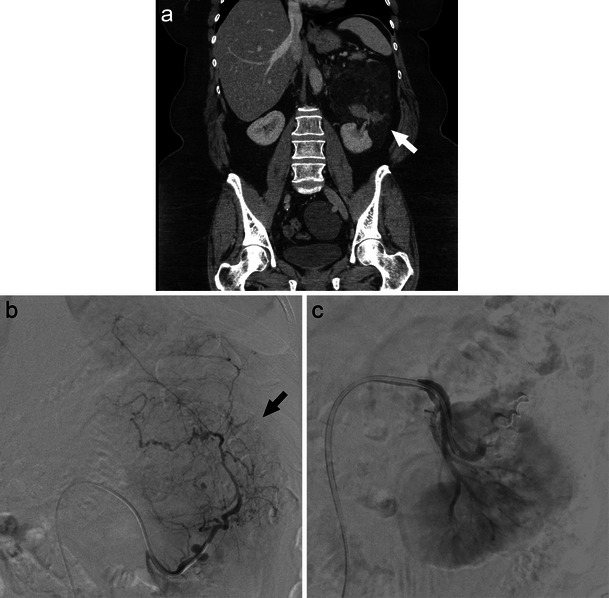
Large left renal AML as seen on CT (**a**, white arrow) and angiography (**b**, black arrow) subsequently embolised with sclerosing agent (ethanol) (**c**)

The most prevalent benign renal tumour is angiomyolipoma (AML), which is composed of fat, vascular, and smooth muscle elements [28]. These benign lesions carry an incidence of 0.02–0.3 % and occur in up to two-thirds of tuberous sclerosis patients [34]. A combination of hypervascularity and inherent vessel weakness in AML leaves these tumours prone to pseudoaneurysm formation and subsequent haemorrhage [28]. Commonly used embolic agents for these lesions include microcoils, nonresorbable particles (polyvinyl alcohol, embospheres), and ethanol [30]. Embolisation by liquid embolic/sclerosing agents, such as ethanol, has been found to be particularly effective in the management of large (>4 cm) AML tumours as well as those associated with haemorrhage or haemodynamic instability (Fig. 8) [28, 30]. Technically, selective TCE is successful in up to 90 % of cases with function preservation in nearly all patients [35]. Despite therapy, however, tumour recurrence may be as high as 30 % [36].

Post-embolisation syndrome—consisting of flank pain, fever, nausea or vomiting, and leukocytosis 24–72 h post-procedure—is the most common complication following TCE, occurring in over 90 % of patients and generally resolving with conservative symptomatic management [30]. Less frequently, coil migration, non-target embolisation, and impairment of renal function may occur following TCE.

## Renovascular trauma

Renovascular injury is relatively uncommon, the majority of cases resulting from blunt trauma or iatrogenic causes [37]. Three-quarters of traumatic renal artery injuries are minor (contusions or superficial lacerations), requiring only conservative management [31].

Renovascular injury secondary to trauma may result in haemorrhage, devascularisation, or urinary tract laceration. Regardless of the suspected injury, CT with intravenous contrast enhancement is the test of choice in the evaluation and diagnosis of renovascular trauma [31]. Occasionally, emergent angiography with embolisation in major renal vascular disruption may be both diagnostic and therapeutic.

**Fig. 9:**
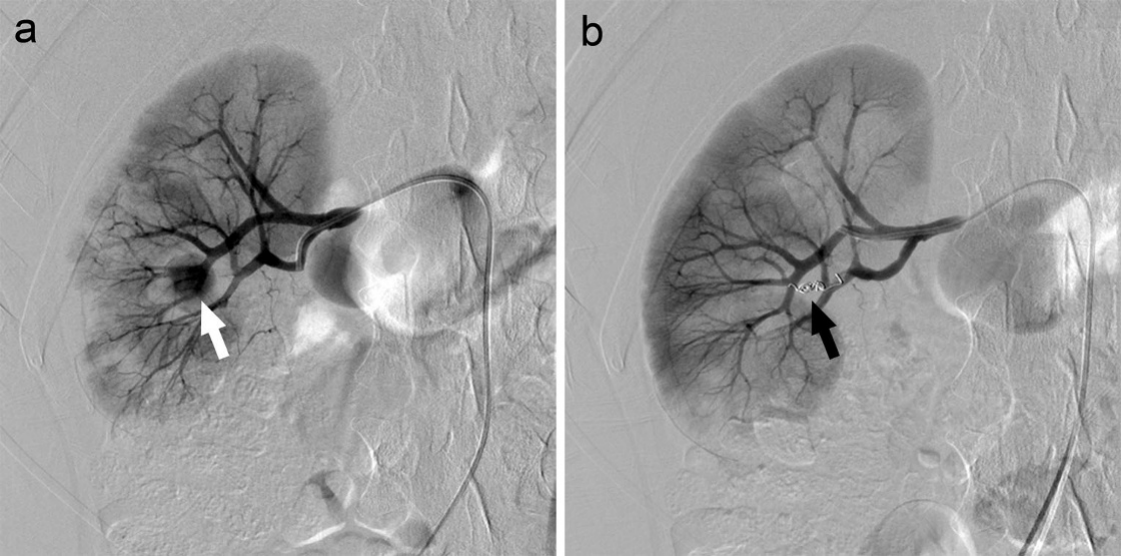
Right renal trauma with persistent haemorrhage of an interlobar artery following a cycling accident demonstrated by angiography (**a**, white arrow). Therapeutic management consisted of coil embolisation of the injured vessel (**b**, black arrow)

Therapeutic management is implemented in cases of high-grade injuries, including those involving persistent haemorrhage and/or haemodynamic instability. Prompt diagnosis and management are crucial as prolonged periods (>3 h) of renal ischaemia may result in severe tubular necrosis and renal dysfunction [31]. In these instances, coil embolisation with the aim of decreasing or arresting haemorrhage is the principle means of therapy (Fig. 9). With embolisation, care should be taken to minimise or avoid nontarget infarction and preserve renal function by selectively targeting the injured vasculature. Entire-organ embolisation by Gelfoam, particles or vascular plugs is generally reserved for cases of trauma to non-functioning kidney [38].

Haemorrhage control by embolisation has been shown to be successful in 84–100 % of renal trauma cases [31, 38]. As outlined earlier, complications from embolisation include renal infarction, iatrogenic vascular injury, and post-embolisation syndrome [38].

Renal artery dissection, avulsion, or laceration secondary to trauma may result in devascularisation. Traumatic dissection develops secondary to intimal tearing and subsequent thrombus formation. Endovascular stenting can be used to restore arterial flow in these cases. Nephrectomy or early surgical vascular reconstruction is avulsion and laceration given propensity for significant haemorrhage [29].

Renal artery pseudoaneurysms, AVFs, arteriocalyceal fistulas, and urinomas are rare lesions that may also develop secondary to renal trauma. Arteriocalyceal fistulas often present as either gross or persistent haematuria following penetrating trauma or iatrogenic injury to vascular and collecting systems. These lesions are typically amenable to selective renal artery embolisation [27].

## Resistant hypertension

Resistant hypertension (RH) is defined as “blood pressure that remains above goal in spite of concurrent use of three antihypertensive agents of different classes” or hypertension requiring four or more medications for effective management [39]. RH affects 9 % of all hypertensive patients and 13 % of those receiving antihypertensive therapy [40]. These patients are at an increased risk for adverse cardiovascular events.

Various factors, including renal artery stenosis, have been implicated in the development of RH. Physiologically, RH has been associated with elevated activity in both the efferent and afferent renal sympathetic nerves. Fittingly, renal denervation is an emerging endovascular treatment modality in the management of this disease [41, 42].

The denervation procedure implements the delivery of catheter-based radiofrequency ablation through the main renal artery. Thermal energy generated in this process acts to effectively disrupt the sympathetic nerves in the adventitia, thus decreasing vascular tone. While studies are ongoing, suggested indications for renal denervation include patients with uncontrolled systolic BP (>160 mmHg; >150 mmHg in diabetics) despite optimised lifestyle factors and antihypertensive medications (>3 synergistic drugs) [43].

Studies comparing the effectiveness of renal sympathetic denervation to standard medical therapy have demonstrated contradictory results. Early nonrandomised studies and randomised, unblinded trials have shown significant blood pressure reduction in denervation patients (BP reduction of 33/11 mmHg at 6 months) with no evidence of decline in kidney function, renal artery stenosis, or aneurysmal dilatation [41, 42]. A more recent blinded, randomised trial that incorporated a sham control procedure and a significantly larger patient population did not demonstrate a significant reduction of systolic blood pressure at 6-month follow-up [44].

## Conclusion

The role of endovascular intervention in the management of renovascular disease is vast and inevitably expanding. As outlined in this review, a variety of minimally invasive procedures have been employed to treat renal artery aneurysm and pseudoaneurysm, renal arteriovenous malformations and fistulas, renal tumours, and renovascular trauma in a safe and effective manner. Endovascular intervention in renal artery stenosis remains debatable, with indications in specific circumstances, and unclear in circumstances of resistant hypertension.
